# Application of Cox Proportional Hazards Model in Case of Tuberculosis Patients in Selected Addis Ababa Health Centres, Ethiopia

**DOI:** 10.1155/2014/536976

**Published:** 2014-01-12

**Authors:** Kabtamu Tolosie, M. K. Sharma

**Affiliations:** Addis Ababa University, Addis Ababa, Ethiopia

## Abstract

*Introduction*. Tuberculosis (TB) is a chronic infectious disease and mainly caused by mycobacterium tuberculosis (MTB). It has been one of the major causes of mortality in Ethiopia. The objective of the study was to identify factors that affect the survival of the patients with tuberculosis who started treatment for tuberculosis. *Methods*. This was a retrospective study in six randomly selected health centres in Addis Ababa, Ethiopia. The data were obtained from medical records of TB patients registered from September 2012 to August 2013 and treated under directly observed treatment surgery (DOTS) strategy. Kaplan Meier plots, logrank tests, and Wilcoxon tests were used to assess the survival pattern. Cox proportional hazards model for multivariable analysis was discussed. *Results*. Out of the total 826 registered TB patients, 105 (12.71%) died during the study period and 712 (87.29%) were censored. Based on Kaplan Meier survival curves, logrank test, and Wilcoxon test, it was found that the patients had statistically significant differences in survival experience with respect to age, body weight at initiation of treatment, TB patient category, and HIV status. Multivariable Cox hazards regression analysis revealed that the covariates age, TB patient category, HIV, and age by HIV interaction were significant risk factors associated with death status in TB patients. *Conclusion*. Deaths of individuals with diseases especially HIV coinfected and nonnew TB cases were high. Therefore, this needs to strengthen the follow-up of patients with TB treatment from the day of anti-TB treatment initiation to completion days.

## 1. Introduction

Tuberculosis (TB) is the leading cause of death from a single bacterial species among adults around the world. The World Health Organization (WHO) estimated that one-third of the world population is infected with *Mycobacterium tuberculosis*, with 9.4 million new cases and 1.3 million deaths in 2009 [1].

Reducing death, eliminating disease, and preventing the development of drug-resistant TB are the major goals of TB control [2]. TB-related death is often referred to as a TB control indicator [3].

Although the developed countries in Europe and North America have well-equipped treatment facilities and provide free and sufficient anti-TB drugs, treatment success rates there are still below WHO's goal of 85%, which may be because of the relatively high death rates. In those countries, TB cases are found in the relatively higher age group and are associated with comorbidities [4].

In previous studies, even in developed countries with good national TB control programs, more than 10% of cases died during the follow-up period. In high-resource settings, but not in most high TB-burden countries, most cases of death during TB treatment are often because of causes other than TB [5].

On March 24, 1882, Dr. Robert Koch announced the discovery of *Mycobacterium tuberculosis*, the bacteria that cause tuberculosis (TB). Dr. Koch's discovery was the most important step taken toward the control and elimination of this deadly disease. In 1982, a century after Dr. Koch's announcement, the first World TB Day was sponsored by the World Health Organization (WHO) and the International Union against Tuberculosis and Lung Disease (IUATLD). The event was intended to educate the public about the devastating health and economic consequences of TB, its effect on developing countries, and its continued tragic impact on global health [6].

Tuberculosis (TB) has been one of the major causes of morbidity and mortality in Ethiopia for long. Accordingly, the Ethiopian Ministry of Health and its stakeholders have put their unreserved and integrated efforts in this health problem. Among these efforts is the well developed Health Management Information System (HMIS) for Tuberculosis programs. However, the direction where Tuberculosis in Ethiopia is heading has not been well analyzed and unpackaged by epidemiologically relevant factors. So, what are the factors that affect the survival of TB patients? It might be gender, age, TB patients category, type of TB, smear result, HIV, and body weight at initiation of treatments of patients. Therefore, to fill this gap there is a need to study the factors that are affecting the survival of patients with tuberculosis. The objectives of this study are (i) to identify the factors that are affecting the survival of the patients with tuberculosis, (ii) to estimate survival time probabilities of the TB patients, (iii) to compare the survival probabilities of the TB patients with respect to different risk factors, and (iv) to identify the factors influencing death status of patient by using the Cox proportional hazards model.

## 2. Data and Methodology

### 2.1. The Data

The data was a retrospective cohort study based on TB patients that were registered in unit TB registers in the health facilities providing DOTS in six randomly selected Addis Ababa Governmental health centres, Addis Ababa, Ethiopia. In this study we used secondary data which was collected from patient follow-up records. Information for this study was extracted from documents of all TB cases registered from September 2012 to August 2013 in six DOTS clinics located in six randomly selected health centres.

### 2.2. Variables in the Study

The explanatory (independent) variables of interest in this study include demographic factors disease, and medicine related factors, and characteristics of the disease. The response (dependent) variable is continuous; it is length of time of treatment for tuberculosis patients.

### 2.3. The Response Variable

The dependent variable or response is the waiting time until the occurrence of an event (dead: 1, alive or censored: 0). Observations are censored, in the sense that, for some units, the event of interest has not occurred at the time the data are analyzed.

### 2.4. Predictor Variables

Predictors or explanatory variables which are called covariates are those whose effect on the waiting time we wish to assess. The predictor (covariate) variables which are assumed to influence the survival of TB patients included in the model are (i) age, (ii) gender, (iii) TB patients category, (iv) type of TB, (v) smear result, (vi) initial weight of patients, and (vii) HIV status.

## 3. Method of Data Analysis

### 3.1. Survival Analysis

The study focused on time to event (time to death by tuberculosis), so the appropriate method of this particular study was survival analysis. We have used Kaplan-Meir estimator and Cox proportional hazard model for the analysis and model building. We have also used logrank tests and Wilcoxon tests for comparison of survival functions. Kaplan Meier analysis was used to study survival pattern; the KM plot, which is a step function, gives some indications about the shape of the survival distribution. The figure in general shows if the pattern of one survivorship function lies above another which means the group defined by the upper curve lived longer, or had a more favourable survival experience, than the group defined by the lower curve.

### 3.2. The Proportional Hazards Model

It was used for multivariate analysis to identify factors associated with death from tuberculosis and Cox proportional hazards (PH) model given by

(1) $$\begin{array}{c} \lambda \left(t\;|\;z\right)=\lambda_{0}\left(t\right)e^{Z^{T}\beta }, \end{array}$$

where **Z**  =  (*Z*_1_,…,*Z*_*p*_)^*T*^  and  **β**  =  (*β*_1_,…,*β*_*p*_)^*T*^, **Z** is a *p* × 1 vector of covariates such as treatment indicators and prognostic factors, and **β** is a *p* × 1 vector of regression coefficient.

The parameter was estimated by using partial likelihood functions. We used three different tests to assess the significance of the coefficients in Cox proportional hazards model: the partial likelihood ratio test, the Wald test, and the score test.

### 3.3. Selection of Covariates

We used Hosmer and Lemeshow [7] and Collett [8] that recommended the procedure in variable selection, including all variables that are significant in the univariable analysis at the 20 to 25 percent level and also any other variables which are presumed to be clinically important to fit the initial multivariable model.

### 3.4. Overall Goodness of Fit

To assess the overall goodness of fit of a Cox proportional hazards regression model Arjas [9] suggests plotting the cumulative observed versus the cumulative expected number of events for subjects with observed (not censored) survival times. If the model fit is adequate, then the points should follow a 45-degree line beginning at the origin.

## 4. Results

The statistical packages SAS and STATA have been used to analyze the data.

### 4.1. Summary Statistics

Out of the total 826 registered TB patients 105 (53 male and 52 female) or 12.71% died during the study period and 712 (87.29%) were censored. The age group (≥45 years) showed the highest percentage (18.3%) with respect to death proportions among the other two age groups. In TB patient category nonnew case had higher percentage (21.93%) of death. The percentages of death 10.4%, 13.99% and 12.99% occurred in the patients with pulmonary positive, pulmonary negative, and extrapulmonary types of TB, respectively, and the patients with positive smear result had lower death percentage. HIV-positive TB patients are the highest risk group for death, that is, 22.18%. Patients with body weight at initiation of treatment (≥35 kg's) had lower risk group for death (Table 1).

### 4.2. Descriptive Survival Analysis

Table 2 exhibits that out of 826 TB patients, 721 patients were censored (87.29%) and 105 patients died (12.71%). The median follow-up time was 168 days for patients that are censored (range from 15 to 284 days); 25% of the patients had 176 days of follow up (upper quartile). The median time of death was 52 days (range from 1 to 190 days). This shows that most of the events/deaths occurred in the earlier months of anti-TB treatment.

**Figure 1:**
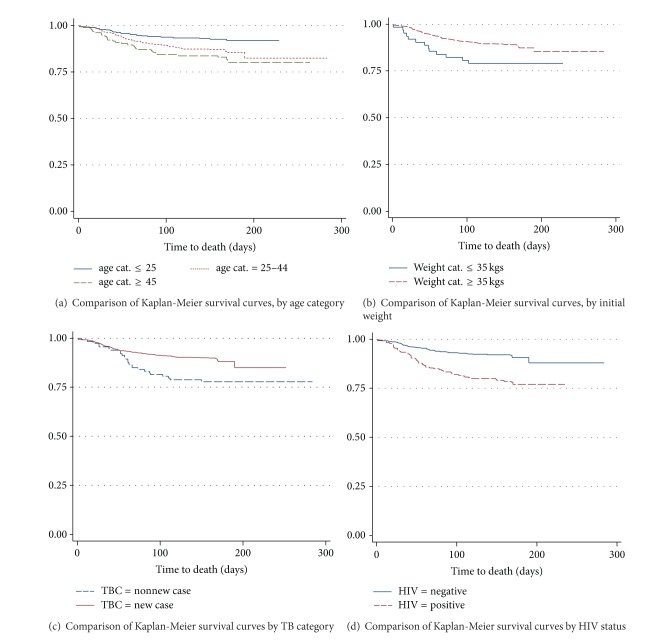
The plot of the estimate of Kaplan-Meier survivor curves of TB patients under DOTS in AA health centers (a) age category, (b) initial weight of patients, (c) TB patient category, and (d) HIV status.

Figure 1 exhibits that there were differences among survivor curves of age category, initial weight, TB patient category, and HIV status for TB patients. However, there were not clear differences among survivor curves of gender, smear result, and type of TB.

Based on Table 3, we find that logrank test and Wilcoxon test are not significant in survival experience between the various categories of gender, smear result, and type of TB. But, they are significant in survival experience of the patients in different categories of age, body weight at initiation of treatment, TBC, and HIV status (at *α* = 5%).

### 4.3. Results of the Cox Proportional Hazards Model

We begin with a multivariable model that contains all variables which were significant in the univariate Cox proportional hazard model at the 20–25 percent level.

Table 4 exhibits the summary of seven covariate variables in the univariate analysis. The most appropriate subset of these predictors will be selected in the multivariable model based on their contribution to the maximized log partial likelihood of the model (−2LL). The highest reduction in $-2\text{LL}(\hat{\beta })$ is observed for HIV status. This difference is 24.599 which is statistically significant (*P*  value < 0.0001) and suggests that an improvement over the null model would be achieved by including HIV status. The reduction in $-2\text{LL}(\hat{\beta })$ on adding TBC to the null model is 8.19, which is significant. The next reduction in $-2\text{LL}(\hat{\beta })$ on adding age of patients to the null model is 6.728, which is also significant. By using the Wald chi-Square test, the variable age, smear result, TB category, and HIV status are significant at the 25-percent level and therefore they are candidates for inclusion in multivariable model. Age, TB Category, and HIV status have relatively strong associations with the death of TB patients. Omitting the predictors or covariates gender, initial weight, and type of TB from the model does not bring significant changes in the value of $-2\text{LL}(\hat{\beta })$. Therefore, these predictors become the first to be removed from the multivariable models.

The next step is to fit the multivariable Cox proportional model that contains age, smear result, TBC, and HIV status. So, at this stage we have a multivariable model which includes the three main effect covariates age, TB category (TBC), and HIV status (HIV). These covariates are significant at 5% level of significance.

The other important step is considering variables that are nonsignificant at univariable analysis but may be confounders. The effect of adding each of the three variables gender, weight, and types of TB (TTB) in the model is shown in Table 5. In particular, when gender, initial weight, and TTB are added, the increase in $-2\text{LL}(\hat{\beta })$ is nonsignificant values, that is, 0.228, 2.142, and 0.736, respectively.

The final step in the model building process is the consideration of interaction terms shown in Table 6. The Wald test was used to assess the significance of reasonable and possible interactions. The decision for rejection of the null hypothesis is −2LL_2_- (−2LL_1_) > *χ* ^2^ (*α* = 0.05) = 3.84. Table 6 shows that the interaction HIV × age was significant. And this is an indication that the interaction of HIV and the age of the patient affects the survival time of the patient.

The results shown in Table 6 ensure that the preliminary model of the study will contain three main effects and one interaction effect. Now, all covariates are significant at 5% level of significance (Table 7). The next step is to check the linearity of continuous covariates in the preliminary multivariable model.

### 4.4. Checking for Linearity of Continuous Covariates in the Model

**Figure 2:**
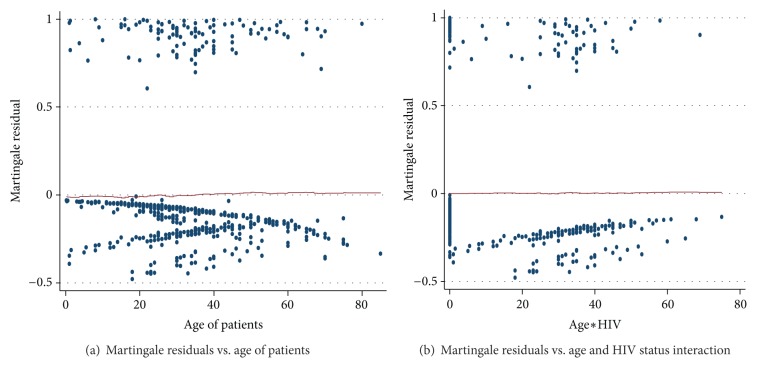
Plots of Martingale residuals computed for continuous covariates of TB patients under DOTS in AA health centers (a) age and (b) age and HIV status interaction.

It can be seen that the plots of martingale residuals in Figure 2 are random showing no systematic patterns or trends, and the LOESS smoothed curve appears to lie about a horizontal line through zero, supporting the inclusion of the untransformed covariates age and age and HIV interaction have a linear relationship with the survival time in Cox model.

After a preliminary model has been fitted to an observed set of survival data, our next step would be to assess the adequacy of the fitted model. The model will not be identified as the final model until its fit and adherence to model assumptions.

### 4.5. Diagnosis of the Mode

#### 4.5.1. Assessment of the Proportional Hazards Assumption

The results of tests of all the time-dependent variables in Table 8 were not significant either individually or collectively, so we do not have enough evidence to reject proportionality assumption of all covariates at 5% level of significance.

**Figure 3:**
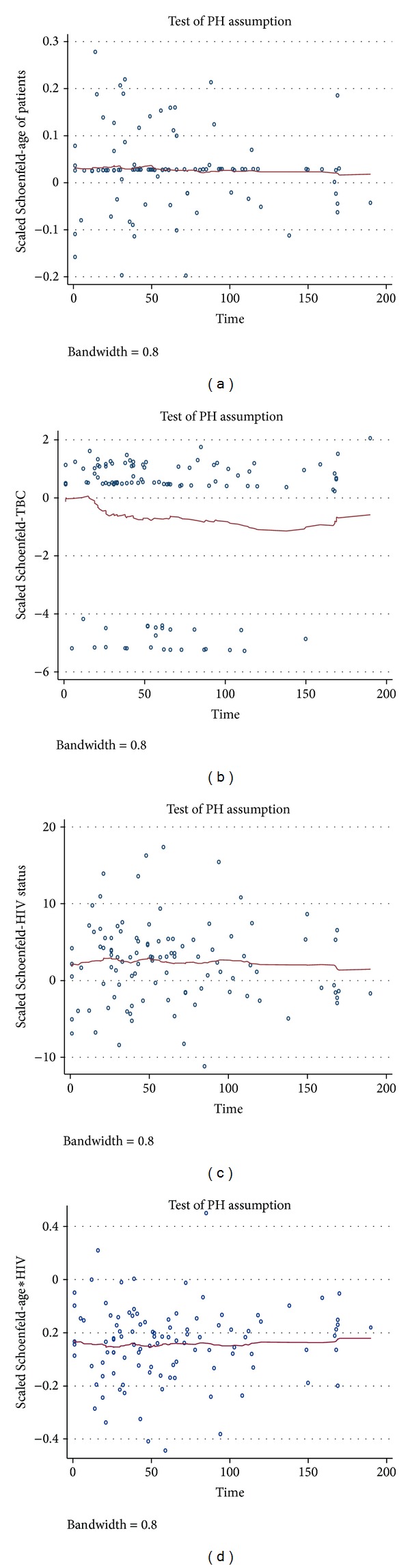
Graphs of the scaled Schoenfeld residuals and their LOESS smooth curves for the covariates: (a) age of patients, (b) TB patient category, (c) HIV status, and (d) age and HIV status interaction.

The plots of the scaled Schoenfeld residuals and the lowness smooth curves shown in Figures 3(a)–3(d) support the assumption of proportional hazards for each of the four covariates. That is, each subplot in the figure is random, smooth and approximates a horizontal through zero or slope approximately equal to zero. This indicates that none of the four covariates had interaction with log of time; also the plots support the proportional hazards assumption.

#### 4.5.2. Identification of Influential and Poorly Fit Data

**Figure 4:**
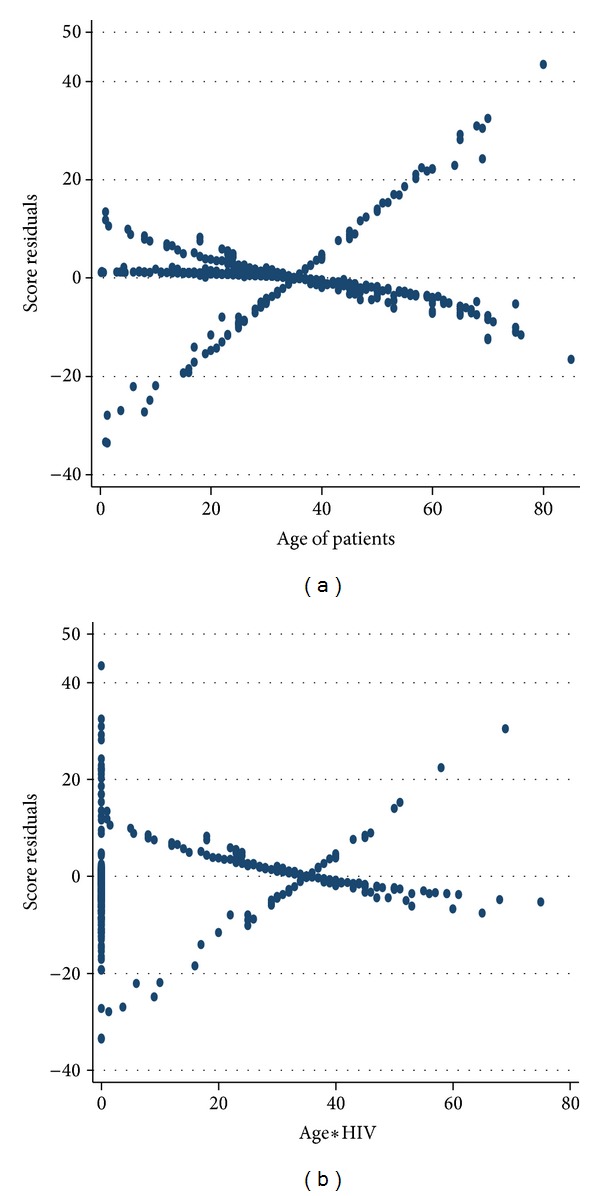
Graphs of the score residuals computed from the model in Table 7 for (a) age of TB patients and (b) age by HIV status interaction.

The score residuals for age in Figure 4(a) display fan shape with the smallest distance near the mean age of 35 and increasing in absolute value for ages increasingly older or younger than 35. The purpose of the plot is to see whether there are subjects whose ages yield unexpectedly large values. This would be seen in the graph as a point lying well away from the others in the plots. In the figure there is one point in the top right at age 80 that falls a bit away from the rest of the points. However, the distance between this point and the others is not striking. The oldest subject, age 85, has score residuals that are well within range of values. Thus, we conclude that there are no high leverage values for age of TB patients.

**Figure 5:**
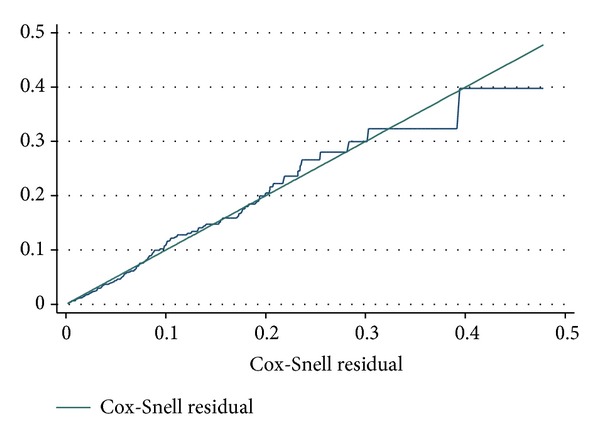
Cumulative hazard plot of the Cox-Snell residuals of the proportional hazards Cox regression model in Table 7. The 45-degree straight line through the origin is drawn for reference.

The score residuals for age × HIV interaction are plotted in Figure 4(b). The plots have one point in the top left corner that fall a bit away from the rest of the other points. But the distance between these points and the others is not striking. The two subsets, one in the top right and one in the bottom right, have score residuals that are well within range of values. Thus, we conclude that there are no high leverage values for age by HIV interaction. In general, the plots in Figure 5 have shown that there are no strikingly large score residuals.

The first six largest changes in parameter estimates are shown in Table 9. To begin with the largest difference for covariate age is observed for patient numbered 730. The result exhibits that the change in the parameter estimate, if the data for this patient is discarded, is 0.0023234. The standard error, of the parameter estimate for age in the full data set is 0.0076. That is, the percentage change in parameter estimate if the observation is removed is about 30.57% of the standard error that is, less than one standard error. Thus, removing this observation would not bring a significant change on age of patients. It is similar for covariates TBC, HIV, and age by HIV interaction. Therefore, it can be concluded that there was no aberrant observation in the data set that illegitimately inflated the estimates of the parameters of the covariates in the final model.

#### 4.5.3. Checking for Overall Goodness of Fit

The cumulative hazard plot of the Cox-Snell residuals is shown in Figure 5. We see that the hazard function is reasonably straight line that has a unit slope and zero interception. It approximates the 45-degree line very closely except for very large values of time. Overall we would conclude that the final model fits the data very well. Therefore, the model with estimates as given in Table 7 is the final model.

#### 4.5.4. Interpretation of the Results

The results of the fitted final model in Table 7 are interpreted in terms of hazard ratios (HR). The coefficient of the categorical covariates is interpreted as the logarithm of the ratio of the hazard of death to the baseline (reference group) hazard. That is, they are interpreted by comparing the reference group with others. Similarly, the coefficient for a continuous explanatory variable indicates the estimated change in the logarithm of the hazard ratio for a unit increase in the value of the respective covariate when the remaining covariates in the model are controlled.

Only the covariate TB patient category (TBC) has hazard ratio that is estimated by exponentiating its estimated coefficient. This is because the covariates age and HIV are involved in interaction. The estimated coefficient for a new case of TB was −0.54298, which decreases the hazards of experiencing death by a factor exp(−0.54289) = 0.581  (95% CI: 0.369–0.916); that is, the patients with a new case of TB have about 41.9% lower mortality rate than patients with nonnew case TB. The 95-percent confidence interval suggested that the rate could be as much as 63.1 percent lower to only 8.4 percent lower. The estimated hazard ratio pointed to a significant benefit for the new case of the two TB patient categories, controlling for all other model covariates.

Age and HIV status are present in the model, with both main effects and their interaction. Since HIV status is at two levels, we present hazard ratios for age at each HIV status rather than for HIV status at each age. For example, the estimate hazard ratios for an increase of 15 years of age at HIV-negative and HIV-positive were found to be 1.51, 0.82, respectively. This means that being older by 15 years at HIV-negative increased the rate of death by about 51 percent and HIV-positive reduces the rate of death by about 18 percent.

## 5. Discussion

This study is an attempt to identify the factors that affect the survival of the patients with tuberculosis; we found that from the Kaplan-Meier survival estimates there was a significant difference in survival by the age, body weight at initiation of treatment, TB patient category, and HIV status. However, there were no differences among survival curves of gender, smear result, and type of TB patients. The logrank test also showed that there was no significant difference in survival experience between the various categories of gender, smear result, and type of TB (*P*  value > 0.05). However, the test showed that the survival experience of patients in different categories of age (logrank statistic = 11.0320,  *P*  value = 0.0053), initial weight (logrank statistic = 4.4974,  *P*  value = 0.0179), TBC (logrank statistic = 9.7252, *P*  value = 0.0008), and HIV status (logrank statistic = 27.6614, *P*  value ≤ 0.0001) differ significantly. The multivariable Cox proportional hazards regression results analysis indicated that the three covariates age, TB patient category, HIV, and the interaction age by HIV were significantly associated with death among TB patients. Gender, body weight at initiation of treatment, smear result, and type of TB were not significantly associated with factors that are affecting the survival of patients with tuberculosis.

A study by Oursler et al. [5] showed that a total of 29 (21%) of the 139 patients died during treatment; the median time to death among these patients was 39 days and follow up for survivors was 202 days. (Lo et al. [10]) showed that, 50% of deaths occurred within 2 months. In this study we found that out of 826 TB patients, 721 patients were censored (87.29%) and 105 patients died (12.71%) during treatment; the median follow-up time is 168 days for patients that are censored (range from 15 to 284 days), 25% of the patients had 176 days of follow up (upper quartile). The median time to death among those patients who died was 52 days (ranges from 1 to 190 days); this shows that most of the events/deaths occurred in the earlier months of TB treatment initiation.

The survival curves of TB patient among age groups are significantly different. There was no difference in the survival curves of male and female patients and according to type of disease [11]. A study by Getahun et al. [12] conducted in Addis Ababa showed that the survival status was significantly different between patient age, weight at initiation of anti-TB treatment, patient category, year of enrolment, and treatment centre. We also found a similar result, no differences among survivor curves of gender and type of disease. However, there was a difference among survivor curves of age category, body weight at initiation of treatment, TB patient category, and HIV status for TB patients.

Age has been identified as an important risk factor for death in tuberculosis patients. Different studies showed that age was a factor that is affecting the survival of TB patients. According to Horne et al. [13] in Washington State, mortality was independently associated with increasing age. A study in Maryland, community-based cohort of patients with drug-susceptible pulmonary TB, showed age was strongly associated with the risk of death [5]. A study in Brazil showed that age was statistically significant in the multivariable Cox regression model [14, 15]. Another study also showed that age has been identified as an important risk factor for death in tuberculosis patients [10–12, 16–18] (Lo et al.). Our study also found that age was statistically a significant risk factor for death.

A study was carried out in Brazil aimed at analyzing survival probability and identifying risk factors for death from tuberculosis in a cohort of patients living in Recife who started treatment for tuberculosis. During follow up HIV-positive was statistically significant in the multivariable Cox regression model [14]. Also HIV coinfection was statistically associated with an increased risk of death in TB patients during treatment [5, 13, 15, 19]. We have also found a similar result; that is, HIV coinfection was a statistically significant risk factor for death in TB patient, and this means the risk of death in TB patients with HIV infection was higher than in those without HIV infection (hazard ratio = 9.888 *P*  value ≤ 0.0001) in multivariable Cox proportional hazard regression model.

Similar to our finding, other studies have also shown that TB patient category (TBC) was statistically associated with death of patients with tuberculosis [14, 15, 20, 21] (Mathew et al.).

The present study identified that body weight at initiation of treatment was not the risk factor for death in tuberculosis patients in multivariable Cox proportional regression model during antituberculosis treatment period (hazards ratio = 0.991, *P*  value = 0.2659). However, studies by [12, 14, 20] reported that body weight at initiation of treatment was a risk factor for death from TB and is associated with survival of patients who begin treatment for tuberculosis.

In this study, the covariates gender and type of TB (TTB) were not found to be factors that affect the survival of patients with TB. But, different studies reported that males were at higher risk factor for death in TB patients [13, 16, 19, 22, 23] (Low et al.) and types of tuberculosis (positive pulmonary, negative pulmonary, and extrapulmonary tuberculosis) were identified as the significant factors for mortality of tuberculosis patients [15, 18, 19].

In this study age with HIV interaction was found a risk factor associated with death of tuberculosis patients. This study is consistent to the study conducted by (Lo et al.) [10]; the study revealed that HIV infection is a significant factor among younger age groups of tuberculosis patients.

Limitations of the study are as follows. The study is conducted based on secondary data which might have incomplete and biased information. Also information might have been missed in case of many censored observations. In many tuberculosis patients, multiple causes of death may act simultaneously, so the cause of death may not be determined accurately.

## Conflict of Interests

The authors declare that there is no conflict of interests regarding the publication of this paper.

## Figures and Tables

**Table 1:** Characteristics of tuberculosis patient data under DOTS from six randomly selected governmental health centers in Addis Ababa, Ethiopia, from September 2011 to August 2012.

Patients characteristics	Summary of the number of death and censored values				
	Total	Death	Percent death	Censored	Percent censored
Gender					
Male	414	53	12.80	361	87.20
Female	412	52	12.62	360	87.38
Age categories					
0–24	284	22	7.75	262	92.25
25–44	389	55	14.14	334	85.86
≥45	153	28	18.30	125	81.70
TB patients cat.					
New	712	80	11.24	632	88.76
Nonnew	114	25	21.93	89	78.07
Type of TB					
Pul. positive	202	21	10.40	181	89.60
Pul. negative	293	41	13.99	252	86.01
Extra pulmonary	331	43	12.99	288	87.01
Smear result					
Positive	202	21	10.4	181	89.60
Negative	624	84	13.46	540	86.54
HIV status					
Positive	239	53	22.18	186	77.82
Negative	587	52	8.86	535	91.14
Initial weight					
<35	62	13	20.97	49	79.03
≥35	764	92	12.04	672	87.96

**Table:** (a)

Status of TB patients		
Status	Frequency	Percent
Censored	721	87.29
Dead	105	12.71

**Table:** (b)

Status of patients	*N*. Obs.	Mean	Std. Dev.	Median	Lower quartile	Upper quartile	Min.	Max.
Censored	721	170.791	18.291	168.000	168.000	176.000	15.000	284.000
Dead	105	63.867	47.012	52.000	29.000	88.000	1.000	190.000

**Table 3:** Results of the Logrank test, Wilcoxon test and −2log (LR) for the categorical variables of TB patients under DOTS in six randomly selected AA health centers.

Test of no difference in survival over strata							
Covariates	DF	Logrank		Wilcoxon		−2log (LR)	
		Chi-Square	Pr > Chi-Square	Chi-Square	Pr > Chi-Square	Chi-Square	Pr > Chi-Square
Gender	1	0.0020	0.9641	0.0167	0.8973	0.0011	0.9740
Age	2	11.0320	0.0040	10.4640	0.0053	11.5554	0.0031
Weight	1	4.4974	0.0339	5.6019	0.0179	3.9437	0.0470
Smear res.	1	1.6355	0.2009	1.7743	0.1828	1.7244	0.1891
TBC	1	9.7252	0.0018	11.3382	0.0008	8.4141	0.0037
TTB	2	1.8631	0.3939	2.0941	0.3510	1.9208	0.3827
HIV	1	27.6614	<0.0001	28.9458	<0.0001	25.6999	<0.0001

**Table 4:** Results of the univariable proportional hazards Cox regression model of TB patients DOTS in six randomly selected AA health centers.

Analysis of maximum likelihood estimates								
Covariates	DF	Parameter estimate	Standard error	−2LL	Lik. ratio *P* value	Score *P* value	Wald *P* value	Hazard ratio
Gender	1	−0.00878	0.19526	1382.970	0.9641	0.9641	0.9641	0.991
Age	1	0.01626	0.00608	1376.244	0.0095	0.0072	0.0074	1.016
Weight	1	−0.00859	0.00772	1381.761	0.2711	0.2665	0.2659	0.991
Smear result	1	−0.31097	0.24441	1381.251	0.1896	0.2012	0.2033	0.733
TBC	1	−0.70235	0.22993	1374.782	0.0042	0.0018	0.0023	0.495
TTB cat. II	1	0.26331	0.26657	1381.039	0.3805	0.3944	0.3233	1.301
TTB cat. III	1	0.36374	0.26882				0.1760	1.439
HIV	1	0.98580	0.19524	1358.373	<0.0001	<0.0001	<0.0001	2.680
The value of −2LL for the null model is 1382.972								

**Table 5:** Result of partial likelihood ratio test for those variables not significant in the univariable analysis fitted by being included in the model containing those variables significant in multivariable analysis one at a time.

Variables	−2LL ($\hat{\beta }$)	−2LL ($\hat{\beta }$) difference	DF	Pr > ChiSq
Age + TBC + HIV	1349.058			
Age + TBC + HIV + gender	1348.830	0.228	1	0.633
Age + TBC + HIV + weight	1346.916	2.142	1	0.143
Age + TBC + HIV + TTB	1348.302	0.736	2	0.692

**Table 6:** Result of partial likelihood ratio test for the contribution of the interaction effect.

Model fit statistics				
Interactions	−2LL_2_ with main effects only	−2LL_1_ with main and interaction effects	−2LL_2_- (−2LL_1_)	Sig.
Age, TBC	1368.802	1368.189	0.613	Do not reject
Age, HIV	1353.644	1345.312	8.332	Reject
TBC, HIV	1353.368	1350.085	3.283	Do not reject

**Table:** (a)

Testing global null hypothesis: bETA = 0			
Test	Chi-Square	DF	Pr > ChiSq
Likelihood ratio	42.6252	4	<0.0001
Score	45.1009	4	<0.0001
Wald	40.3081	4	<0.0001

**Table:** (b)

Analysis of maximum likelihood estimates								
Covariates	DF	Parameter estimate	Standard error	Chi-Square	Pr > ChiSq	Hazard ratio	95% HR Conf. limit	
Age	1	0.02745	0.00760	13.0603	0.0003	1.028	1.013	1.043
TBC	1	−0.54298	0.23231	5.4629	0.0194	0.581	0.369	0.916
HIV	1	2.29131	0.50215	20.8209	<0.0001	9.888	3.695	26.457
Age ∗ HIV	1	−0.04028	0.01354	8.8468	0.0029	0.961	0.935	0.986
The value of −2LL for the model is 1340.347								

**Table:** (a)

Analysis of maximum likelihood estimates						
Covariates	DF	Parameter estimate	Standard error	Chi-Square	Pr > ChiSq	Hazard ratio
Age	1	0.01919	0.02212	0.7526	0.3857	1.019
TBC	1	0.20274	0.95433	0.0451	0.8318	1.225
HIV	1	1.09680	1.13372	0.9359	0.3333	2.995
AgHIV	1	−0.02914	0.01722	2.8638	0.0906	0.971
Age ∗ log (time)	1	0.00223	0.00561	0.6904	0.6904	1.002
TBC ∗ log (time)	1	−0.19525	0.23829	0.6714	0.4126	0.823
HIV ∗ log (time)	1	0.31882	0.27050	1.3891	0.2385	1.375
agHIV ∗ log (time)	1	−0.0001785	0.0001725	1.0712	0.3007	1.000

**Table:** (b)

Linear hypotheses testing results			
Label	Wald Chi-Square	DF	Pr > ChiSq
Test proportionality	2.1579	4	0.7067

**Table:** (a)

Deleted Obs. (*i*)	$\Delta_{i}{\hat{\beta }}_{1}$	% change parameter of the std. error	Deleted Obs. (*i*)	$\Delta_{i}{\hat{\beta }}_{2}$	% change parameter of the std. error
730	0.0023234	30.57	413	−0.0439987	18.94
162	−0.0021205	27.90	585	−0.0428435	18.44
262	−0.0020957	27.58	343	−0.0427599	18.41
421	−0.0017624	23.20	368	−0.0423473	18.23
542	0.0017002	22.37	544	−0.04214	18.14
580	0.001611	21.20	447	−0.041601	17.91
Sd. error (${\hat{\beta }}_{1}$) of full model = 0.00760			Sd. error (${\hat{\beta }}_{2}$) of full model = 0.23231		

**Table:** (b)

Deleted Obs. (*i*)	$\Delta_{i}{\hat{\beta }}_{3}$	% change parameter of the std. error	Deleted Obs. (*i*)	$\Delta_{i}{\hat{\beta }}_{4}$	% change parameter of the std. error
133	0.1185484	23.61	548	0.0042068	31.07
548	−0.1155908	23.02	63	0.0032745	24.18
204	0.1146464	22.83	133	−0.0031662	23.38
244	−0.1056003	21.03	204	−0.0030298	22.38
162	−0.1008921	20.10	244	−0.0027001	19.94
262	−0.098443	19.60	282	−0.0024873	18.37
Sd. error (${\hat{\beta }}_{3}$) of full model = 0.50215			Sd. error (${\hat{\beta }}_{4}$) of full model = 0.01354		
